# Interactions Between Plasma Amyloid and Aging Markers to Determine Clinically Meaningful Cognitive Decline

**DOI:** 10.1093/geroni/igab046.2607

**Published:** 2021-12-17

**Authors:** Wan-Hsuan Lu, Kelly Giudici, Bruno Vellas, Philipe Souto de Barreto

**Affiliations:** CHU Toulouse: Centre Hospitalier Universitaire de Toulouse, Gérontopôle de Toulouse, Institut du Vieillissement, Midi-Pyrenees, France

## Abstract

Background: Brain amyloidosis is a well-known pathological hallmark of Alzheimer’s disease (AD) and can be early identified by measuring plasma amyloid-β (Aβ) status. Growing evidence implicates the biological mechanisms of aging, including chronic inflammation, mitochondrial dysfunction and neurodegeneration, in AD pathogenesis. This study aims to investigate the interactions between plasma Aβ status and aging markers on clinically meaningful cognitive decline. Methods: This secondary analysis from Multidomain Alzheimer Preventive Trial (MAPT) enrolled 401 community-dwelling older adults (mean age ± SD: 76.7 ± 4.6 years) who had clinical dementia rating (CDR) scale as 0 or 0.5, and who had their plasma biomarkers measured: amyloidosis: Aβ42/40 ratio; inflammatory: tumor necrosis factor receptor type 1 (TNFR-1), interleukin-6 (IL-6), monocyte chemoattractant protein-1 (MCP-1), C-reactive protein (CRP); mitochondrial dysfunction: growth differentiation factor 15 (GDF-15); neurodegeneration: neurofilament light chain (NfL). Cognitive decline was determined by diagnosed dementia and worsening CDR status. Cox regression and moderation modeling were applied to examine the interrelationships between biomarkers and risk of cognitive decline. Results: Among 401 participants, 43.9% were cognitive normal (CDR=0) and 56.1% were mild cognitive impairment (CDR=0.5) initially. After 3.3 ± 1.1 years of follow-up, 7.0% of population evolved dementia and 34.2% had worsening CDR status. GDF-15 and NfL presented prospective associations with incident dementia. However, risk of dementia associated with plasma Aβ did not change after considering the serum level of GDF-15 and NfL. Conclusion: The markers of mitochondrial dysfunction and neurodegeneration did not partially explain the associations between plasma Aβ status and cognitive decline in older adults.

